# 3-(1*H*-Tetra­zol-5-yl)pyridinium 3-(2*H*-tetra­zol-5-yl)pyridinium dinitrate

**DOI:** 10.1107/S160053680901839X

**Published:** 2009-06-27

**Authors:** Li-Jing Cui

**Affiliations:** a Ordered Matter Science Research Center, College of Chemistry and Chemical Engineering, Southeast University, Nanjing 210096, People’s Republic of China

## Abstract

In the title compound, C_6_H_6_N_5_^+^·NO_3_^−^, there are two different isomers of the cation within the asymmetric unit. The dihedral angles between the the pyridinium and tetra­zole rings are 2.54 (15) and 13.36 (18)° in the two cations. In the crystal, the packing of ions is stabilized by N—H⋯O and N—H⋯(O,O) hydrogen bonds, forming clusters composed of four ion pairs.

## Related literature

For background to tetra­zole derivatives, see: Dai & Fu (2008 ▶); Wang *et al.* (2005 ▶); Wen (2008 ▶); Xiong *et al.* (2002 ▶).
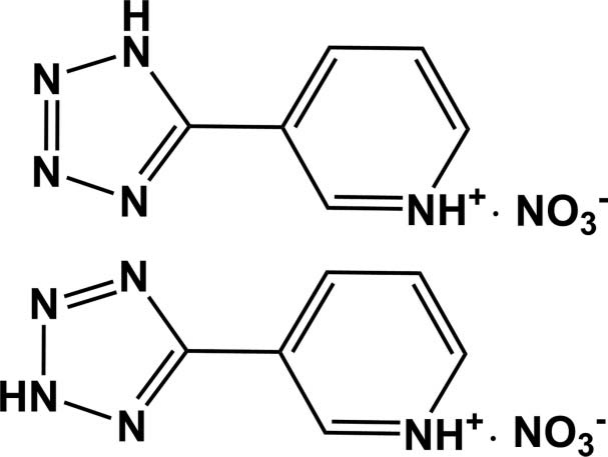


## Experimental

### 

#### Crystal data

C_6_H_6_N_5_^+^·NO_3_^−^
*M* *_r_* = 210.17
Triclinic, 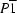

*a* = 6.9157 (14) Å
*b* = 10.575 (2) Å
*c* = 13.346 (3) Å
α = 110.10 (3)°
β = 100.65 (3)°
γ = 95.87 (3)°
*V* = 886.2 (3) Å^3^
*Z* = 4
Mo *K*α radiation
μ = 0.13 mm^−1^
*T* = 298 K
0.35 × 0.30 × 0.15 mm

#### Data collection

Rigaku Mercury2 diffractometer
Absorption correction: multi-scan (*CrystalClear*; Rigaku, 2005 ▶) *T*_min_ = 0.956, *T*_max_ = 0.981
9175 measured reflections
4035 independent reflections
2272 reflections with *I* > 2σ(*I*)
*R*_int_ = 0.045

#### Refinement

*R*[*F* ^2^ > 2σ(*F* ^2^)] = 0.057
*wR*(*F* ^2^) = 0.150
*S* = 1.03
4035 reflections
279 parameters
H atoms treated by a mixture of independent and constrained refinement
Δρ_max_ = 0.18 e Å^−3^
Δρ_min_ = −0.22 e Å^−3^

### 

Data collection: *CrystalClear* (Rigaku, 2005 ▶); cell refinement: *CrystalClear*; data reduction: *CrystalClear*; program(s) used to solve structure: *SHELXS97* (Sheldrick, 2008 ▶); program(s) used to refine structure: *SHELXL97* (Sheldrick, 2008 ▶); molecular graphics: *SHELXTL* (Sheldrick, 2008 ▶); software used to prepare material for publication: *SHELXTL*.

## Supplementary Material

Crystal structure: contains datablocks I, global. DOI: 10.1107/S160053680901839X/hb2945sup1.cif

Structure factors: contains datablocks I. DOI: 10.1107/S160053680901839X/hb2945Isup2.hkl

Additional supplementary materials:  crystallographic information; 3D view; checkCIF report

## Figures and Tables

**Table 1:** Hydrogen-bond geometry (Å, °)

*D*—H⋯*A*	*D*—H	H⋯*A*	*D*⋯*A*	*D*—H⋯*A*
N9—H9*A*⋯O2^i^	0.93 (2)	1.80 (3)	2.700 (3)	164 (2)
N2—H2*A*⋯O1^ii^	0.88 (3)	2.16 (3)	2.998 (3)	161 (2)
N2—H2*A*⋯O2^ii^	0.88 (3)	2.16 (3)	2.890 (3)	140 (2)
N5—H5*A*⋯O4	0.86	1.94	2.791 (3)	168
N10—H10*A*⋯O4	0.86	2.06	2.891 (3)	163
N10—H10*A*⋯O6	0.86	2.19	2.873 (3)	137

Symmetry codes: (i) 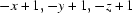; (ii) 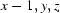.

**Fig. 1.**
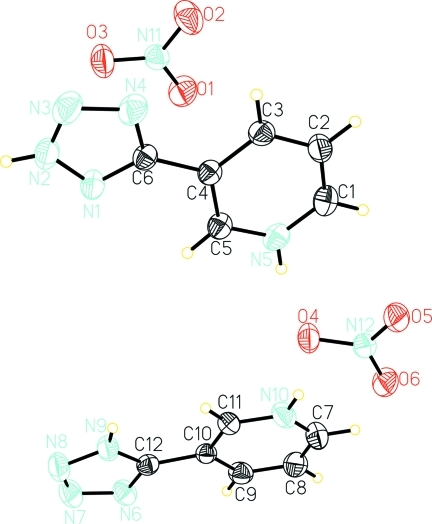
A view of (I) with displacement ellipsoids drawn at the 30% probability level.

**Fig. 2.**
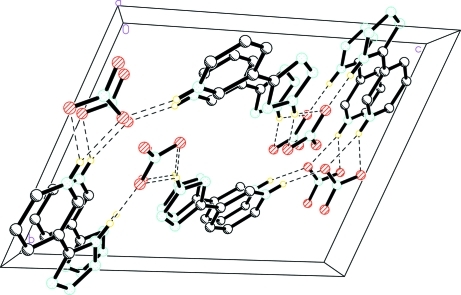
The crystal packing of (I) viewed along the a axis showing the two-dimensional hydrogen bonding network. H atoms not involved in hydrogen bonding (dashed lines) have been omitted for clarity.

## supplementary crystallographic information

### Comment

Tetrazole derivatives have found wide range of applications in coordination chemistry because of their multiple coordination modes as ligands to metal ions and for the construction of novel metal-organic frameworks (Wang, *et al.* 2005; Xiong, *et al.* 2002; Wen 2008). We report here the crystal structure of the title compound, 3-(1*H*-tetrazol-5-yl)pyridinium 3-(2*H*-tetrazol-5-yl)pyridinium nitrate (Fig. 1).

The title compound contains two different isomers of the cation, one with the H atom attached to the N2 and the other with the H atom attached to N9. Each isomer is built up by two different rings. The pyridinium and the tetrazole rings are nearly coplanar and only twisted from each other by a dihedral angle of 2.54 (15)° [13.36 (18)° for the second molecule]. The geometric parameters of the tetrazole rings are comparable to those in related molecules (Wang *et al.* 2005; Dai & Fu, 2008).

The packing of ions is stabilized by N—H···O hydrogen bonds, to form a zero-dimentional sheets parallel to the (1 0 0) plane that is composed of four pairs of ions (Table 1, Fig. 2).

### Experimental

Picolinonitrile (30 mmol), NaN _3_ (45 mmol), NH_4_Cl (33 mmol) and DMF (50 ml) were added in a flask under nitrogen atmosphere and the mixture stirred at 383 K for 20 h. The resulting solution was then poured into ice-water (100 ml), and a white solid was obtained after adding HCl (6 *M*) till pH = 6. The precipitate was filtered and washed with distilled water. Colourless blocks of (I) were obtained from the crude product by slow evaporation of an ethanol/HNO_3_ (50:1 *v*/*v*) solution.

### Refinement

The tetrazole-ring H atoms were located in a difference map and freely refined. The other H atoms were fixed geometrically (C–H = 0.93 Å and N–H = 0.86 Å) and refined as riding with *U*_iso_(H) = 1.2*U*_eq_(C or N).

### Crystal data

**Table:**

C_6_H_6_N_5_^+^·NO_3_^−^	*Z* = 4
*M**_r_* = 210.17	*F*(000) = 432
Triclinic, *P*1	*D*_x_ = 1.575 Mg m^−^^3^
Hall symbol: -P 1	Mo *K*α radiation, λ = 0.71073 Å
*a* = 6.9157 (14) Å	Cell parameters from 4035 reflections
*b* = 10.575 (2) Å	θ = 3.1–27.5°
*c* = 13.346 (3) Å	µ = 0.13 mm^−^^1^
α = 110.10 (3)°	*T* = 298 K
β = 100.65 (3)°	Block, colourless
γ = 95.87 (3)°	0.35 × 0.30 × 0.15 mm
*V* = 886.2 (3) Å^3^	

### Data collection

**Table:**

Rigaku Mercury2 diffractometer	4035 independent reflections
Radiation source: fine-focus sealed tube	2272 reflections with *I* > 2σ(*I*)
graphite	*R*_int_ = 0.045
Detector resolution: 13.6612 pixels mm^-1^	θ_max_ = 27.5°, θ_min_ = 3.1°
CCD profile fitting scans	*h* = −8→8
Absorption correction: multi-scan (*CrystalClear*; Rigaku, 2005)	*k* = −13→13
*T*_min_ = 0.956, *T*_max_ = 0.981	*l* = −17→17
9175 measured reflections	

### Refinement

**Table:**

Refinement on *F*^2^	Primary atom site location: structure-invariant direct methods
Least-squares matrix: full	Secondary atom site location: difference Fourier map
*R*[*F*^2^ > 2σ(*F*^2^)] = 0.057	Hydrogen site location: inferred from neighbouring sites
*wR*(*F*^2^) = 0.150	H atoms treated by a mixture of independent and constrained refinement
*S* = 1.03	*w* = 1/[σ^2^(*F*_o_^2^) + (0.0621*P*)^2^ + 0.11*P*] where *P* = (*F*_o_^2^ + 2*F*_c_^2^)/3
4035 reflections	(Δ/σ)_max_ < 0.001
279 parameters	Δρ_max_ = 0.18 e Å^−^^3^
0 restraints	Δρ_min_ = −0.22 e Å^−^^3^

### Special details

**Table:**

Geometry. All e.s.d.'s (except the e.s.d. in the dihedral angle between two l.s. planes) are estimated using the full covariance matrix. The cell e.s.d.'s are taken into account individually in the estimation of e.s.d.'s in distances, angles and torsion angles; correlations between e.s.d.'s in cell parameters are only used when they are defined by crystal symmetry. An approximate (isotropic) treatment of cell e.s.d.'s is used for estimating e.s.d.'s involving l.s. planes.
Refinement. Refinement of *F*^2^ against ALL reflections. The weighted *R*-factor *wR* and goodness of fit *S* are based on *F*^2^, conventional *R*-factors *R* are based on *F*, with *F* set to zero for negative *F*^2^. The threshold expression of *F*^2^ > σ(*F*^2^) is used only for calculating *R*-factors(gt) *etc*. and is not relevant to the choice of reflections for refinement. *R*-factors based on *F*^2^ are statistically about twice as large as those based on *F*, and *R*- factors based on ALL data will be even larger.

### Fractional atomic coordinates and isotropic or equivalent isotropic displacement parameters (Å^2^)

**Table:**

	*x*	*y*	*z*	*U*_iso_*/*U*_eq_	
O4	0.6923 (3)	0.42367 (18)	0.16340 (15)	0.0676 (5)	
N12	0.7836 (3)	0.3552 (2)	0.09506 (16)	0.0538 (5)	
O5	0.8211 (3)	0.24401 (18)	0.09476 (15)	0.0736 (5)	
N5	0.6669 (3)	0.30395 (19)	0.31822 (16)	0.0562 (5)	
H5A	0.6691	0.3510	0.2770	0.067*	
O6	0.8282 (3)	0.40305 (19)	0.02794 (15)	0.0719 (5)	
N6	0.3306 (3)	1.0327 (2)	0.16715 (18)	0.0631 (6)	
C3	0.6604 (3)	0.1553 (2)	0.44515 (18)	0.0521 (6)	
H3	0.6577	0.1028	0.4885	0.063*	
N1	0.2697 (3)	0.3533 (2)	0.53331 (16)	0.0581 (5)	
C5	0.5298 (3)	0.3174 (2)	0.37811 (18)	0.0499 (6)	
H5	0.4387	0.3755	0.3745	0.060*	
C12	0.3716 (3)	0.9175 (2)	0.17583 (18)	0.0478 (5)	
C6	0.3778 (3)	0.2557 (2)	0.51234 (17)	0.0466 (5)	
N9	0.2512 (3)	0.8800 (2)	0.23196 (17)	0.0578 (5)	
C4	0.5260 (3)	0.2439 (2)	0.44512 (17)	0.0446 (5)	
C10	0.5230 (3)	0.8456 (2)	0.13170 (17)	0.0438 (5)	
N2	0.1669 (3)	0.3194 (3)	0.59678 (17)	0.0641 (6)	
N4	0.3414 (3)	0.1670 (2)	0.56134 (18)	0.0687 (6)	
N7	0.1816 (3)	1.0644 (2)	0.2193 (2)	0.0723 (6)	
C11	0.5523 (3)	0.7245 (2)	0.14313 (19)	0.0524 (6)	
H11	0.4782	0.6892	0.1821	0.063*	
C1	0.8009 (4)	0.2213 (3)	0.3189 (2)	0.0607 (7)	
H1	0.8951	0.2166	0.2768	0.073*	
N3	0.2040 (4)	0.2098 (3)	0.61492 (19)	0.0750 (7)	
C2	0.7973 (4)	0.1445 (2)	0.3817 (2)	0.0577 (6)	
H2	0.8872	0.0849	0.3818	0.069*	
C9	0.6383 (4)	0.8947 (3)	0.0739 (2)	0.0609 (7)	
H9	0.6247	0.9778	0.0664	0.073*	
N10	0.6860 (3)	0.6578 (2)	0.09869 (18)	0.0673 (6)	
H10A	0.7034	0.5828	0.1079	0.081*	
N8	0.1327 (3)	0.9736 (2)	0.25866 (19)	0.0722 (6)	
C7	0.7937 (4)	0.7011 (3)	0.0409 (2)	0.0765 (8)	
H7	0.8835	0.6496	0.0096	0.092*	
C8	0.7731 (4)	0.8206 (3)	0.0275 (2)	0.0752 (8)	
H8	0.8490	0.8522	−0.0126	0.090*	
N11	0.7936 (3)	0.4430 (2)	0.71227 (17)	0.0552 (5)	
O3	0.6532 (3)	0.4772 (2)	0.75145 (17)	0.0817 (6)	
O2	0.8610 (3)	0.3403 (2)	0.71981 (19)	0.0878 (6)	
O1	0.8712 (3)	0.5025 (2)	0.66120 (17)	0.0809 (6)	
H9A	0.236 (4)	0.802 (3)	0.248 (2)	0.069 (8)*	
H2A	0.075 (4)	0.361 (3)	0.625 (2)	0.073 (9)*	

### Atomic displacement parameters (Å^2^)

**Table:**

	*U*^11^	*U*^22^	*U*^33^	*U*^12^	*U*^13^	*U*^23^
O4	0.0790 (12)	0.0713 (11)	0.0790 (12)	0.0375 (10)	0.0464 (10)	0.0393 (10)
N12	0.0476 (11)	0.0620 (13)	0.0548 (12)	0.0134 (10)	0.0152 (10)	0.0229 (11)
O5	0.0974 (14)	0.0641 (12)	0.0720 (12)	0.0379 (11)	0.0306 (11)	0.0287 (10)
N5	0.0711 (14)	0.0495 (11)	0.0544 (12)	0.0048 (10)	0.0232 (11)	0.0245 (10)
O6	0.0786 (13)	0.0856 (13)	0.0746 (12)	0.0245 (10)	0.0400 (11)	0.0441 (11)
N6	0.0656 (14)	0.0485 (12)	0.0753 (15)	0.0186 (10)	0.0165 (12)	0.0207 (11)
C3	0.0610 (15)	0.0476 (13)	0.0510 (14)	0.0125 (11)	0.0136 (12)	0.0212 (11)
N1	0.0603 (13)	0.0582 (12)	0.0607 (13)	0.0165 (10)	0.0265 (11)	0.0201 (10)
C5	0.0607 (15)	0.0390 (12)	0.0513 (13)	0.0091 (10)	0.0178 (12)	0.0158 (10)
C12	0.0515 (14)	0.0453 (13)	0.0438 (12)	0.0070 (10)	0.0082 (11)	0.0150 (10)
C6	0.0520 (14)	0.0427 (12)	0.0435 (12)	0.0067 (10)	0.0136 (11)	0.0134 (10)
N9	0.0576 (13)	0.0595 (13)	0.0640 (13)	0.0192 (11)	0.0237 (11)	0.0252 (11)
C4	0.0514 (13)	0.0368 (11)	0.0438 (12)	0.0064 (10)	0.0124 (11)	0.0126 (10)
C10	0.0459 (12)	0.0440 (12)	0.0420 (12)	0.0096 (10)	0.0087 (10)	0.0168 (10)
N2	0.0613 (14)	0.0750 (16)	0.0570 (13)	0.0155 (12)	0.0265 (12)	0.0182 (12)
N4	0.0868 (16)	0.0672 (14)	0.0757 (15)	0.0224 (12)	0.0410 (13)	0.0417 (12)
N7	0.0666 (15)	0.0580 (14)	0.0874 (17)	0.0235 (11)	0.0184 (13)	0.0173 (13)
C11	0.0530 (14)	0.0531 (14)	0.0543 (14)	0.0168 (11)	0.0129 (12)	0.0217 (11)
C1	0.0588 (16)	0.0592 (15)	0.0626 (16)	0.0078 (12)	0.0244 (13)	0.0165 (13)
N3	0.0845 (17)	0.0857 (17)	0.0724 (15)	0.0164 (13)	0.0395 (14)	0.0397 (13)
C2	0.0575 (15)	0.0568 (14)	0.0624 (15)	0.0190 (12)	0.0210 (13)	0.0206 (13)
C9	0.0582 (15)	0.0695 (16)	0.0628 (16)	0.0135 (13)	0.0128 (13)	0.0342 (14)
N10	0.0663 (14)	0.0599 (13)	0.0762 (15)	0.0272 (11)	0.0133 (13)	0.0234 (12)
N8	0.0632 (14)	0.0714 (15)	0.0802 (16)	0.0264 (12)	0.0262 (12)	0.0170 (13)
C7	0.0626 (18)	0.092 (2)	0.0760 (19)	0.0310 (16)	0.0234 (16)	0.0235 (17)
C8	0.0604 (17)	0.111 (2)	0.0685 (18)	0.0207 (16)	0.0271 (15)	0.0427 (18)
N11	0.0512 (12)	0.0589 (13)	0.0604 (13)	0.0121 (10)	0.0160 (11)	0.0260 (11)
O3	0.0753 (13)	0.0926 (14)	0.1060 (15)	0.0389 (11)	0.0537 (12)	0.0489 (12)
O2	0.0878 (14)	0.0793 (13)	0.1334 (18)	0.0386 (11)	0.0536 (13)	0.0643 (13)
O1	0.0872 (14)	0.0878 (13)	0.0980 (15)	0.0216 (11)	0.0476 (12)	0.0565 (12)

### Geometric parameters (Å, °)

**Table:**

O4—N12	1.268 (2)	C10—C11	1.372 (3)
N12—O5	1.229 (2)	C10—C9	1.385 (3)
N12—O6	1.239 (2)	N2—N3	1.304 (3)
N5—C5	1.337 (3)	N2—H2A	0.88 (3)
N5—C1	1.338 (3)	N4—N3	1.318 (3)
N5—H5A	0.8600	N7—N8	1.288 (3)
N6—C12	1.317 (3)	C11—N10	1.324 (3)
N6—N7	1.354 (3)	C11—H11	0.9300
C3—C2	1.370 (3)	C1—C2	1.354 (3)
C3—C4	1.386 (3)	C1—H1	0.9300
C3—H3	0.9300	C2—H2	0.9300
N1—N2	1.315 (3)	C9—C8	1.377 (4)
N1—C6	1.319 (3)	C9—H9	0.9300
C5—C4	1.374 (3)	N10—C7	1.321 (3)
C5—H5	0.9300	N10—H10A	0.8600
C12—N9	1.334 (3)	C7—C8	1.354 (4)
C12—C10	1.450 (3)	C7—H7	0.9300
C6—N4	1.343 (3)	C8—H8	0.9300
C6—C4	1.469 (3)	N11—O3	1.215 (2)
N9—N8	1.345 (3)	N11—O1	1.228 (2)
N9—H9A	0.93 (2)	N11—O2	1.253 (2)
			
O5—N12—O6	122.0 (2)	N3—N2—H2A	118.7 (17)
O5—N12—O4	120.4 (2)	N1—N2—H2A	126.4 (17)
O6—N12—O4	117.6 (2)	N3—N4—C6	105.8 (2)
C5—N5—C1	123.6 (2)	N8—N7—N6	110.6 (2)
C5—N5—H5A	118.2	N10—C11—C10	120.0 (2)
C1—N5—H5A	118.2	N10—C11—H11	120.0
C12—N6—N7	106.2 (2)	C10—C11—H11	120.0
C2—C3—C4	120.4 (2)	N5—C1—C2	119.0 (2)
C2—C3—H3	119.8	N5—C1—H1	120.5
C4—C3—H3	119.8	C2—C1—H1	120.5
N2—N1—C6	101.2 (2)	N2—N3—N4	105.6 (2)
N5—C5—C4	118.8 (2)	C1—C2—C3	119.6 (2)
N5—C5—H5	120.6	C1—C2—H2	120.2
C4—C5—H5	120.6	C3—C2—H2	120.2
N6—C12—N9	108.2 (2)	C8—C9—C10	120.1 (2)
N6—C12—C10	125.2 (2)	C8—C9—H9	120.0
N9—C12—C10	126.7 (2)	C10—C9—H9	120.0
N1—C6—N4	112.5 (2)	C7—N10—C11	123.0 (2)
N1—C6—C4	125.0 (2)	C7—N10—H10A	118.5
N4—C6—C4	122.4 (2)	C11—N10—H10A	118.5
C12—N9—N8	108.6 (2)	N7—N8—N9	106.5 (2)
C12—N9—H9A	129.7 (16)	N10—C7—C8	119.8 (3)
N8—N9—H9A	121.5 (16)	N10—C7—H7	120.1
C5—C4—C3	118.6 (2)	C8—C7—H7	120.1
C5—C4—C6	120.5 (2)	C7—C8—C9	119.2 (3)
C3—C4—C6	120.9 (2)	C7—C8—H8	120.4
C11—C10—C9	117.9 (2)	C9—C8—H8	120.4
C11—C10—C12	120.9 (2)	O3—N11—O1	122.8 (2)
C9—C10—C12	121.2 (2)	O3—N11—O2	120.1 (2)
N3—N2—N1	114.9 (2)	O1—N11—O2	117.1 (2)

### Hydrogen-bond geometry (Å, °)

**Table:**

*D*—H···*A*	*D*—H	H···*A*	*D*···*A*	*D*—H···*A*
N9—H9A···O2^i^	0.93 (2)	1.80 (3)	2.700 (3)	164 (2)
N2—H2A···O1^ii^	0.88 (3)	2.16 (3)	2.998 (3)	161 (2)
N2—H2A···O2^ii^	0.88 (3)	2.16 (3)	2.890 (3)	140 (2)
N5—H5A···O4	0.86	1.94	2.791 (3)	168
N10—H10A···O4	0.86	2.06	2.891 (3)	163
N10—H10A···O6	0.86	2.19	2.873 (3)	137

Symmetry codes: (i) −*x*+1, −*y*+1, −*z*+1; (ii) *x*−1, *y*, *z*.
